# CPT—DMT Correlations on Regional Soils from Croatia

**DOI:** 10.3390/s22030934

**Published:** 2022-01-25

**Authors:** Kristijan Grabar, Stjepan Strelec, Miljenko Špiranec, Filip Dodigović

**Affiliations:** 1 SPP d.o.o., Koprivnička ulica 47, 42000 Varaždin, Croatia; kristijan@spp.hr (K.G.); miljenko.spiranec@icloud.com (M.Š.); 2 Faculty of Geotechnical Engineering, University of Zagreb, Hallerova aleja 7, 42000 Varaždin, Croatia; filip.dodigovic@gfv.unizg.hr

**Keywords:** clay, silts, CPTu, DMT, in-situ tests, correlations

## Abstract

This paper was prepared based on in situ measurements carried out by the authors using the CPTu and DMT static penetration probes. The list of study sites includes seven specific locations in the northern parts of Croatia and one study site on the southern border of the country. The sites were selected based on the criterion of soil type, which falls into the category of soft to firm, slightly over-consolidated silty clays and silty sands. Intermediate soils are prevalent in the wider region, and most engineers deal with them in their everyday practice. For this reason, local characterization is of most importance for engineering purposes. In this investigation, results of in-situ tests are compared in order to validate the quality of the constrained modulus obtained from a CPT test to the one obtained by a DMT flat dilatometer. A comparison was made between the CPT test cone resistance $Q_{t1}$ and two DMT parameters—normalized modulus $E_{D}/{\sigma^{\prime }}_{v0}$ and horizontal stress index $K_{D}$. Dependencies were analyzed for the main soil groups and intermediate data groups. Clay soils were divided into two subgroups based on the identification parameter $I_{D}$, while silty soils were analyzed in three subgroups. The results for each subgroup differed significantly, and the analyses showed deviations from published values, especially for the intermediate soil groups. The usefulness of the application is demonstrated with examples at two sites, showing improvements over the most commonly used formula for the constrained modulus from the CPT test.

## 1. Introduction

The CPTu test is less reliable in determining the soil constrained modulus because the modulus cone factor $\alpha_{M}$ and the overconsolidation ratio *OCR* are difficult to determine. The cone factor for the constrained modulus M varies considerably within the clayey soil and is reported in the literature with values ranging from 1 to 15 [1]. Suggested values depend on the author and the geographic region where the analysis was performed. Intermediate soils, such as silts and clayey sands, are particularly difficult, and there is no reliable modulus cone factor reported for such soils. This presents a major problem for engineers in interpreting data from CPT. It was first recognized by Senneset et al. [2]. The CPT problem in intermediate soils is represented by the cone advancing penetration rate, where silty soils are under partially drained conditions. The partially drained condition at penetration was extensively investigated and confirmed in a large-scale piezocone test campaign on silty soils in Venice (Italy) [3]. The accuracy of the relationships increases the more narrowly the soil categories are defined. Thus, it is obvious that multiple correlations are required for each soil subgroup. This principle was introduced by Senneset et al., who established a linear correlation between constrained modulus and penetration resistance and found that the rate increases with increasing cone resistance in a standard range $q_{t}<5\;\mathrm{MPa}$ of most silty soils [4]. This issue was further investigated by Robertson [5], who incorporated the soil behavior type index $I_{c}$ into a definition of modulus cone factor and defined cone factor for a range of normalized cone resistance, Equation (17). To date, this is the most commonly used formula for calculating the constrained modulus from the CPT test. On the other side, it is well known that standard interpretation methods tend to overestimate the modulus M from the CPT measurement [6]. This motivated the authors of this paper to investigate the reliability of the standard method by comparing it with the results of a more reliable in-situ method, flat dilatometer DMT.

The DMT dilatometer has a good reputation and is praised in the literature for its reliable modulus measurements because it takes into account the stress history. Obtained correlations will be used for determining the DMT constrained modulus based on CPT test results. This is important from several points of view: the DMT test is less frequently available in practice, and the correlations found in the literature are only valid for the main group of soils. To make the conversion more accurate, the correlations are performed for a narrow soil subgroup and for normalized parameters. There is no example of such a detailed comparison. Clayey soil is separated according to ranges $I_{D}$ < 0.33 (clay) and 0.33 <$\;I_{D}\;$< 0.6 (silty clay). Silty soils will be analysed on even more refined subgroup ranges 0.6 <$\;I_{D}$ < 0.8 (clayey silt), 0.8 < $\;I_{D}$ < 1.2 (silt) and 1.2 < $I_{D}$ < 1.8 (sandy silt).

Research results are compared with published values. For the horizontal stress index $K_{D}$, data from Kulhawy and Mayne [7] (Equation (3)) and Robertson [8] (Equation (4)) were compared. The normalized modulus $E_{D}/{\sigma^{\prime }}_{v0}$ was compared with the expression of Mayne and Liao [9] (Equation (6)). The correlations for identification indexes CPT $I_{C}$ and DMT $I_{D}$ were compared to the published equation derived by Robertson [8] (Equation (15)).

The result and the usefulness of the application are verified in examples at two sites. The comparison is performed in vertical constrained modulus profiles. Three lines are compared in the plot, the constrained modulus profile from the measured DMT data (reference data), the profile calculated from the CPT correlations from this work, and the most commonly used empirical correlation published by Robertson [5] (Equation (17)).

## 2. Overview

The cone penetration test (CPT) was developed in the 1960s in the Netherlands and has the advantages of being fast, nearly continuous, economical and having a solid theoretical background. For modern digital piezocones, which can also measure pore pressures, the test procedure is governed by the standard ISO 22476-1:2012. The technical data of the electronic sensors used in CPT Icone digital cone are listed in Table 1. Another in situ probe, the flat plate dilatometer (DMT), was developed in Italy in the 1980s by Professor Silvano Marchetti. The test procedures are described in ASTM standard D 6635-01.

The DMT allows reproducible and simple determination of geotechnical parameters. Unlike CPT, penetration is not continuous, and measurements are taken every 20 cm, making it slower than the CPT. However, the DMT is more suitable than the CPT for the determining of some geotechnical parameters, especially soil compressibility [1,10].

The results of CPT and DMT are used to estimate various geotechnical parameters, of which stiffness is of great importance. In fine-grained soils, the results of both tests provide reliable estimates of undrained shear strength and over-consolidation ratio (OCR). In addition, CPT is reliable in estimating peak friction angle in coarse-grained soils and DMT in one-dimensional constrained modulus for a wide range of soil types [11]. The accuracy of the dilatometer could be checked with the CPTu data, as they are most valuable when used together [12]. The flat dilatometer is specifically designed to determine soil deformation parameters, allowing a direct (in-situ) estimation of the deformation modulus. As numerous studies have shown, the dilatometer is more sensitive to stress history, with the horizontal stress index *K_D_* being an effective indicator [13]. Therefore, it is considered as a reference for deformation characteristics in this article.

This paper compares the records of pairs of adjacent CPT and DMT soundings at eight sites in Croatia to obtain intercorrelations between two in situ probes typical of the local sediments. Robertson [8] published a paper on this subject, with which the data of this study are compared, expression (4). He established a relationship between two in-situ tests using data from one test and extrapolating to the other. This framework is extended for a more refined classification of mixed soil types. Similar studies not using the normalized parameters of CPT have been published [14,15,16] and showed that the estimation of DMT parameters using the results of CPT from the existing regression analyses could have significant variations, which are highly related to the regional soil type. The main reasons for the different responses are the geological history and deposition processes [17]. By developing correlations for soils specific to the territory of the Republic of Croatia, it is possible to improve the existing correlation equations, which is the main objective of this work. Some authors have extended the correlations for other parameters. Mayne [18] extended a CPTu-DMT interrelationship for the pore pressure. The results led him to develop an equivalent NTH method for DMT to acquire effective mechanical soil parameters and an SCE solution for OCR from DMT. Rabarijoely et al. [19] developed nomograms for determining the relative density Dr from DMT data. All of the proposed formulas are local and are yet to be verified for broader application.

Considering the wide span of mixed silt materials ranging from coarse to fine silt and their behavior ranging from sand-like (DMT drained) to clay-like (DMT undrained), it is the intention of this paper to derive correlations for material groups classified as silty soils into four groups (clayey silt, silt, sandy silt and broader group silty mixtures) and as clayey soils into three groups (clay, silty clay and clayey mixtures). An attempt was made to avoid special soil types in the analysis, i.e., soils with microstructure, aged soils or only partially drained silty soils, so-called “niche silt” [20].

Not many correlations between DMT and normalized CPT parameters have been published. Marchetti et al. [21] suggested a correlation between DMT constrained modulus *M′_-DMT-_* and cone resistance—$q_{t}$. Mayne and Liao [9] suggested a relationship between the $I_{D}$ and the friction ratio $F_{r},$ and between $E_{D}$ and $q_{t}$. Mayne [22] suggested a correlation between the basic DMT measurements ($p_{0}$ and $p_{1}$) and the CPTu measurements ($q_{t}$ and $u_{2}$) in soft clays. Marchetti [11] showed that $K_{D}$ is strongly influenced by the *OCR* and proposed that the *OCR* in fine-grained soils can be estimated from the DMT using:

(1) $$OCR={\left(0.5\cdot K_{D}\right)}^{1.56}$$

Kulhawy and Mayne [7] showed that the normalized cone resistance $Q_{t1}$ is also strongly influenced by *OCR* and suggested that *OCR* in fine-grained soils can be estimated from CPT:

(2) $$OCR=0.3\cdot Q_{t1}$$

Combining Equations (1) and (2) gives:

(3) $$K_{D}=0.88\cdot {\left(Q_{t1}\right)}^{0.64}$$

Robertson [8] proposed a correlation based on the observation that the corrected lift-off pressure ($p_{0}$) is equal to the excess pore pressure ($u_{2}$) around the probe in clays:

(4) $$K_{D}=\left(u_{2}-u_{0}\right)/{\sigma^{\prime }}_{v0}=\beta \cdot {\left(Q_{t1}\right)}^{0.95}+1.05$$

where, on average, β=0.3.

Mayne and Liao [9] presented CPT and DMT data from Piedmont residual soils composed of sands to sandy silts and suggested correlations between $E_{D}$ and $q_{t}$ in the form:

(5) $$E_{D}=5\cdot q_{t}$$

where $q_{t}$ is much greater than $\sigma_{v0}$ and, in the normalized form, Equation (5) is:

(6) $$E_{D}/{\sigma^{\prime }}_{v0}=5\cdot Q_{t1}$$

At the study sites, materials with a slightly over-consolidated nature are mainly present to some extent [23]. However, highly structured clays did not agree well with the DMT measurements and were explicitly marked as groups of outliers in the correlation diagrams and excluded from the statistical analyses.

**Figure 1:**
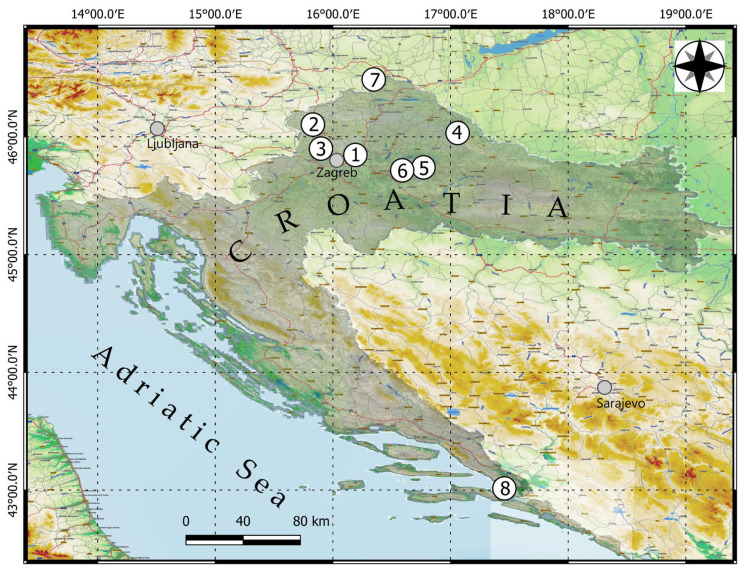
Map of the study sites in the regions of Croatia (numbering of the sites according to the list in Table 2).

The layout of the study sites is shown on the topographic map in Figure 1. The sites are numbered according to the locations listed in Table 2.

## 3. Methods

### 3.1. The Flat Dilatometer Test (DMT)

DMT equipment, application and methodology, as well as original correlations, were developed by Dr. Silvano Marchetti. The hardware consists of a stainless-steel blade with a flat circular steel membrane mounted flush on one side. The single reading consists of reading the values of A and B, which are used to determine the pressures $p_{0}$ and $p_{1}$. The values are corrected for gauge zero offsets, feeler pin elevation and membrane stiffness.

The interpretation sets the three main identifying parameters:

(7) $$\mathrm{Material}\;\mathrm{index}:\;I_{D}=\left(p_{1}-p_{0}\right)/\left(p_{0}-u_{0}\right)$$

(8) $$\mathrm{Horizontal}\;\mathrm{stress}\;\mathrm{index}:\;K_{D}=\left(p_{0}-u_{0}\right)/{\sigma^{\prime }}_{v0}$$

(9) $$\mathrm{Dilatometer}\;\mathrm{modulus}:\;E_{D}=34.7\cdot \left(p_{1}-p_{0}\right)$$

where $u_{0}$ and ${\sigma^{\prime }}_{v0}$ are the pre-insertion in situ equilibrium water pressure and vertical effective stress.

The most significant data obtained from the DMT measurements are the constrained modulus *M′_(DMT)_* values [24], defined as the vertical drained confined (1-D) tangent modulus at ${\sigma^{\prime }}_{v0}.$ It is treated the same as $E_{oed}=1/m_{v}$ obtained by an oedometer. In that context, the dilatometer modulus $E_{D}$ should not be used as such in deformation analyses, but in combination with the $I_{D}$ and $K_{D}$ indexes. The reason for this is primarily because $E_{D}$ does not incorporate information on stress history and lateral pressures. This is, to some degree, incorporated into the $K_{D}$ index. For that reason, the dilatometer modulus $E_{D}$ can be expressed as a combination of $I_{D}$ and $K_{D}$ in the form [10]:

(10) EDσ′v0=34.7·ID·KD

DMT main parameters $I_{D}$ and $K_{D}$ are normalized and dimensionless.

Soil types are identified according to the DMT material index into three main groups: Clays $I_{D}<0.6$; silty mixtures $0.6<I_{D}<1.8$ and sands $I_{D}>1.8$.

The $K_{D}$ parameter could be considered as a lateral earth pressure coefficient ($K_{0}$) at rest, enhanced by the effect of the DMT penetration. Its depth profile is similar in shape to the OCR profile. For normally consolidated clays, the $K_{D}$ value was approximately 2. Several authors have developed correlations between the $K_{D}$ and several geotechnical parameters.

**Figure 2:**
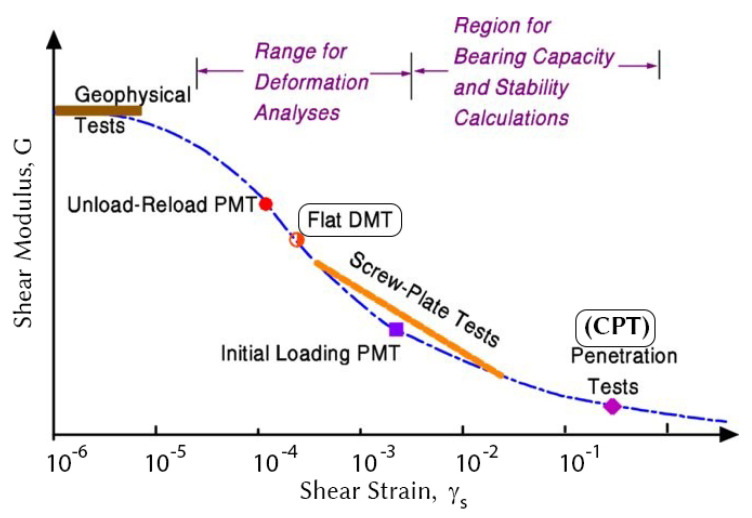
Shear strain for different in-situ probes, Mayne et al., 2001.

At this point, it is necessary to address the strain rate at which the DMT probe stresses the soil. As Mayne et al. [25] has shown, CPT shears the soil at the highest critical shear strain, while the DMT probe shears at a much lower strain, as shown in Figure 2.

The DMT probe strains soil at a level several orders of magnitude lower, which generally measures a higher modulus and is therefore also much more sensitive to changes in soil stiffness [26].

### 3.2. Piezocone Penetration Test (CPT)

The CPT was first introduced in the Netherland in the 1930s as a mechanical test; from the 1960s, it was incorporated with electric strain gauge load cells. The modern CPTu system consists of a digital cone, and because it is capable of measuring pore pressures, it is also called a piezocone. Measurements were conducted with a digital cone manufactured by A.P. van den Berg Netherland named Icone I-CFXYP20-10.

The digital CPT Icone acquisition system consists of a sensor, AD converters, memory and microcontroller, all built into the cone itself. Data transmission from the cone to a digital acquisition box on the surface is entirely digital, so the effect of cables and connectors is negligible. The primary function of the acquisition box is to combine depth information from the rotary encoder mounted on the push cylinder with data from the cone and provide power to the cone’s electronics. The acquisition box is controlled via a laptop through a USB connection and the software package, so the operator is presented with the data from the cone in real-time. A great feature of digital systems becomes apparent when the data transmission between the cone and the acquisition box is interrupted. In this case, all the data of the cone can be retrieved from the memory after the cone has been retracted out of the ground. The icon is calibrated according to ISO 22476-1 Class 2. The technical data of the Icone digital cone are listed in Table 1.

**Figure 3:**
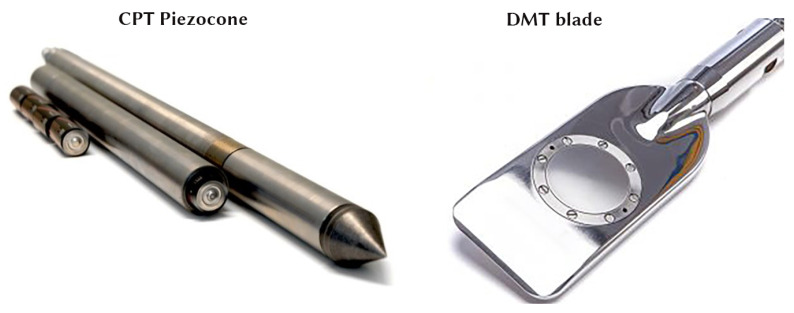
CPT piezocone cone and flat dilatometer DMT blade.

CPT, a cylindrical cone, is thrust into the ground at a velocity of 2 cm/s, continuously measuring the stress at the tip—$q_{c}$, the frictional stress at the sleeve—$f_{s}$, the internal pore pressure—$u_{2}$ (measured behind the cone), and inclination in the x and y axes. Figure 3 shows side-by-side photos of CPT piezocone and DMT blade.

Robertson [27] suggested using the following normalized CPT parameters to identify soil behavior type (SBT):

(11) $$\mathrm{Normalized}\;\mathrm{cone}\;\mathrm{resistance}:\;Q_{t1}=\left(q_{t}-\sigma_{v0}\right)/{\sigma^{\prime }}_{v0}$$

(12) $$\mathrm{Normalized}\;\mathrm{friction}\;\mathrm{ratio}:\;F_{r}=\left[f_{s}/\left(q_{t}-\sigma_{v0}\right)\right]\cdot 100\%$$

(13) $$\mathrm{Pore}\;\mathrm{pressure}\;\mathrm{parameter}:\;B_{q}=\left(u_{2}-u_{0}\right)/\left(q_{t}-\sigma_{v0}\right)=\Delta u/\left(q_{t}-\sigma_{v0}\right)$$

where: $q_{t}=q_{c}+u_{2}\left(1-a\right)$—corrected cone stress (a=0.75; $\sigma_{v0}$—preinsertion in-situ total vertical stress; $u_{2}$—measured pore pressure (position behind the cone); and $\Delta u=\left(u_{2}-u_{0}\right)$—excess penetration pore pressure.

Robertson and Wride [28] presented the boundaries between different soil types using the CPT SBT index $I_{c}$:

(14) $$I_{c}={\left[{\left(3.47-logQ_{t1}\right)}^{2}+{\left(logF_{r}+1.22\right)}^{2}\right]}^{0.5}$$

Soil types are defined for the $I_{c}$ index ranges as follows: clays $I_{c}>2.95$; silt mixtures $2.05<I_{c}<2.95$; sands $I_{c}<2.05$.

The general view is that the estimate of the 1D constrained modulus from CPT undrained cone penetration is considered good, but it can be improved with additional information about the soil [5]. Several other CPT-based indicators can be used to detect subtle differences in soil types [29].

### 3.3. Position and Minimal Distance of CPTu/DMT Pair

The distance between the CPTu and DMT investigation points should be sufficient to avoid interactions. For example, in the standard ISO 22476-1:2012, the distance between the CPTu point and the exploratory borehole is specified to be at least 20 times the borehole diameter. Therefore, the above regulation has been adopted for a 96 mm DMT blade, and a distance of 2 m should generally be sufficient.

## 4. Investigation Data

Eight datasets from CPT-DMT were compiled for a derivation of intercorrelated relationships. The tests were carried out in Croatia at soft clay, clay and silty mixtures. Diluvial clays are composed mainly of calcite, illite minerals and quartz in a mass ratio of 5:2:1 and mainly of Plio-Quaternary and Holocene age. The vertical DMT and CPT profiles were conducted side by side, and the depth of the tests ranged up to 16 m. Table 2 and Table 3 summarize the measured datasets from adjacent CPT and DMT profiles. The tables also show typical ranges of indexes related to the penetration probes.

## 5. Results

### 5.1. Clay-like Soils (I_D_ < 0.6, I_C_ > 2.95)

**Figure 4:**
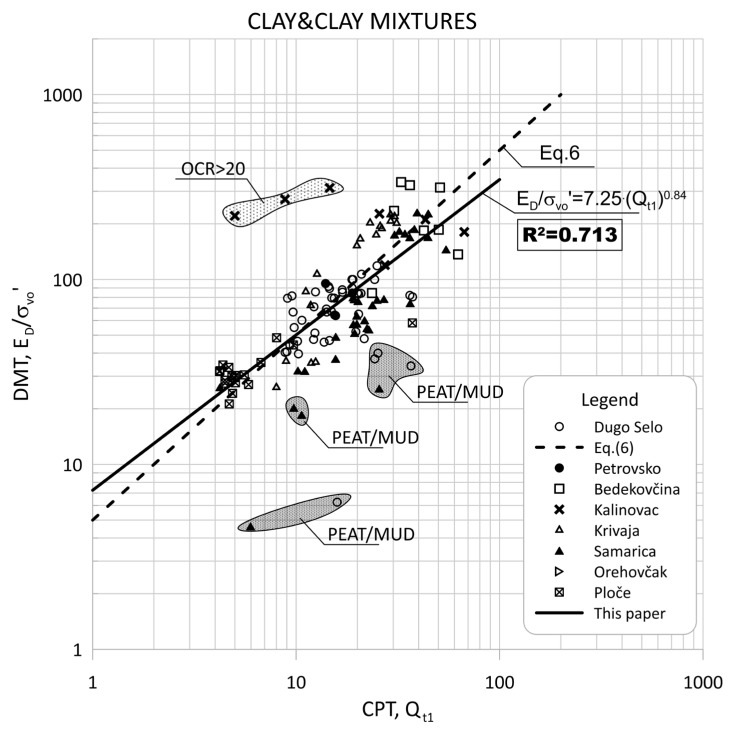
Summary of measured values from adjacent CPT and DMT profiles of $Q_{t1}$ versus $E_{D}/{\sigma^{\prime }}_{v0}$ for clays and clay mixtures ($I_{D}$ < 0.6, $I_{c}$ > 2.95).

**Figure 5:**
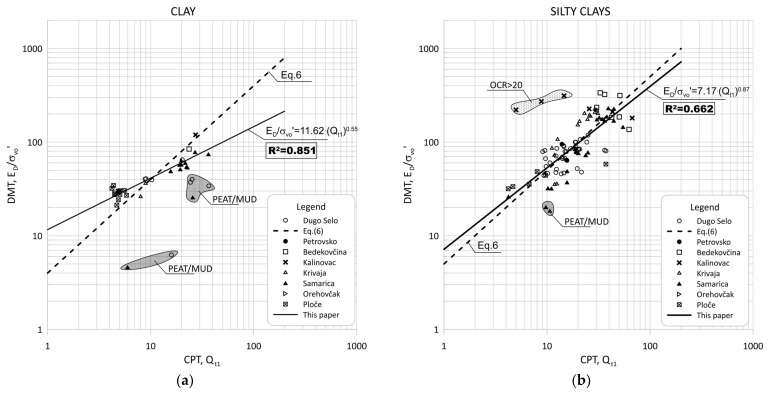
Summary of measured values from adjacent CPT and DMT profiles of $Q_{t1}$ versus $E_{D}/{\sigma^{\prime }}_{v0}$ for: (**a**) clays ($I_{D}$ < 0.33, $I_{c}$ > 2.95); (**b**) silty clays (0.33 < $I_{D}$ < 0.6, $I_{c}$ > 2.95).

Figure 4 shows the data for $Q_{t1}$ versus $E_{D}/{\sigma^{\prime }}_{v0}$ based on measurements made at sites from Table 2 in clays and clay mixtures. Figure 5 shows the data separated by individual groups for clays and silty-clay soil.

The groups of outliers are marked in Figure 4 and Figure 5 because these groups represent small, thin layers within a thicker soil deposit. In addition, thin layers had very high *OCR* values that stand out in the graphs because they are within the predominant soil. Such data were excluded from the statistical analysis.

**Figure 6:**
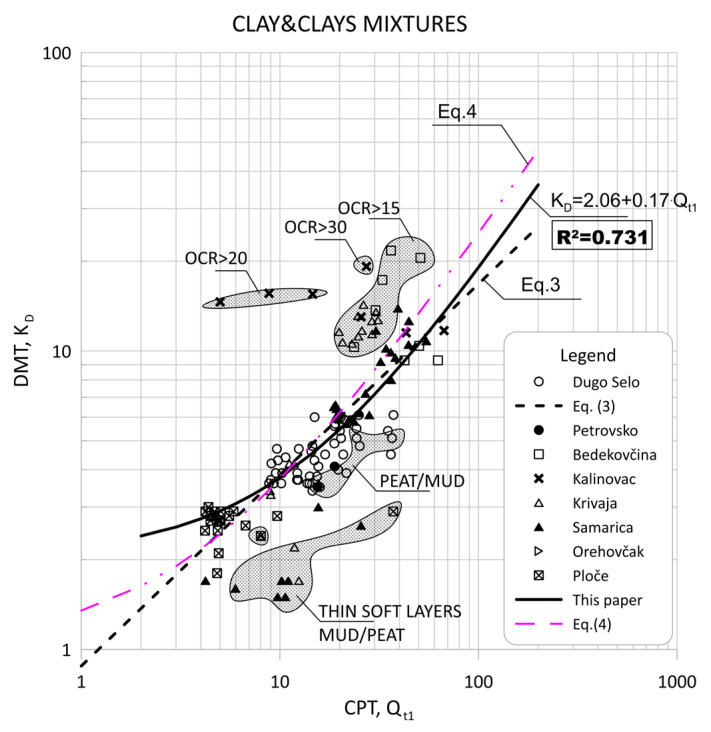
Summary of measured values from adjacent CPT and DMT profiles of $Q_{t1}$ versus $K_{D}$ for clays and clay mixtures ($I_{D}$ < 0.6, $I_{c}$ > 2.95).

**Figure 7:**
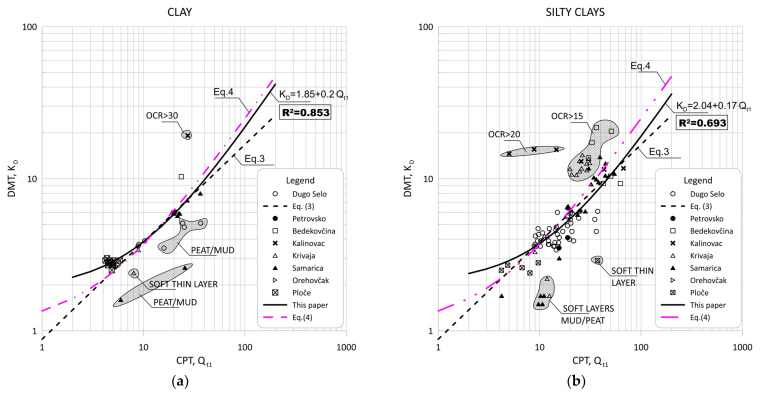
Summary of measured values from adjacent CPT and DMT profiles of $Q_{t1}$ versus $K_{D}$ for: (**a**) clays ($I_{D}$ < 0.33, $I_{c}$ > 2.95); (**b**) silty clays (0.33 < $I_{D}$ < 0.6, $I_{c}$ > 2.95).

Comparisons of CPT normalized parameter $Q_{t1}$ and DMT index $K_{D}$ are shown in Figure 6 and Figure 7.

The scattering of each data group is visible in Figure 5 and Figure 6, which can be divided into two main regions of outliers. The very soft soils (peat/mud—below the regression line) or highly over-consolidated soils (OCR > 15—above the regression line). Some highly OCR soils were thin layers within thicker soil deposits, and some had different mechanical properties and belonged to different materials.

### 5.2. Silty-like Soils (0.6 < I_D_ < 1.8, 2.05 < I_C_ < 2.95)

**Figure 8:**
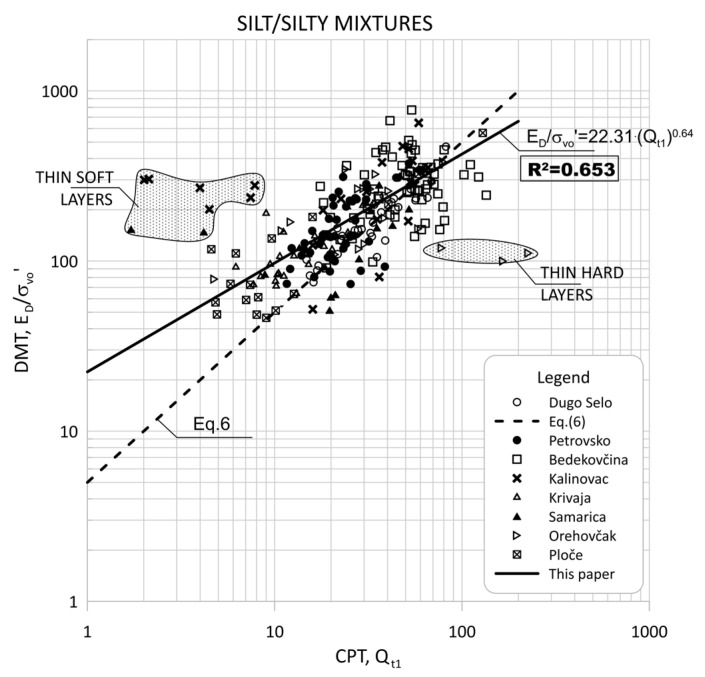
Summary of measured values from adjacent CPT and DMT profiles of $Q_{t1}$ versus $E_{D}/{\sigma^{\prime }}_{v0}$ for silt and silty mixtures (0.6 < $I_{D}$ < 1.8, 2.05 < $I_{c}$ < 2.95).

Figure 8 show the data obtained for $Q_{t1}$ versus $E_{D}/{\sigma^{\prime }}_{v0}$ based on measurement at eight sites for silt and silty mixtures.

**Figure 9:**
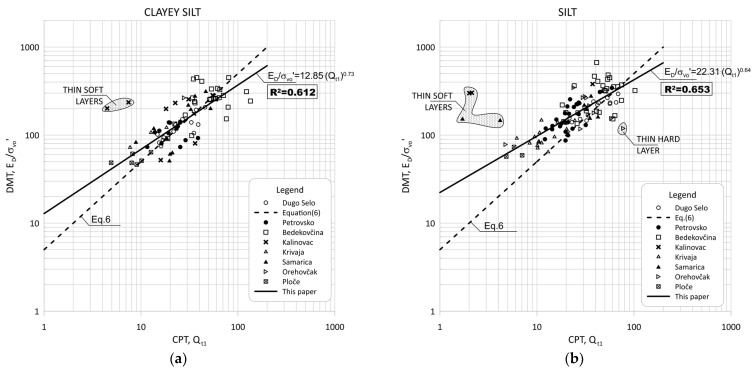
Summary of measured values from adjacent CPT and DMT profiles of $Q_{t1}$ versus $E_{D}/{\sigma^{\prime }}_{v0}$ for: (**a**) clayey silt (0.6 < $I_{D}$ < 0.8, 2.05 < $I_{c}$ < 2.95); (**b**) silt (0.8 < $I_{D}$ < 1.2, 2.05 < $I_{c}$ < 2.95).

**Figure 10:**
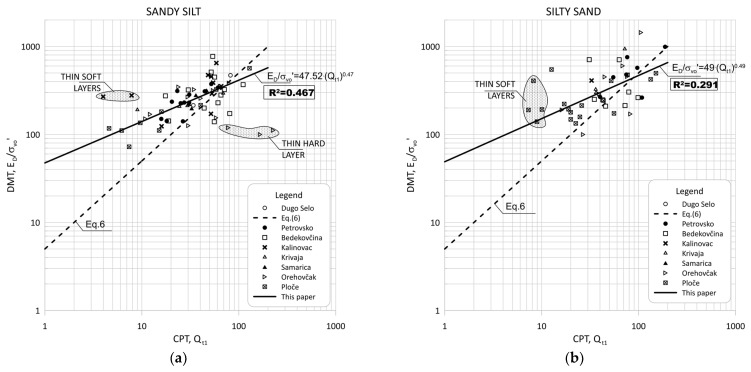
Summary of measured values from adjacent CPT and DMT profiles of $Q_{t1}$ versus $E_{D}/{\sigma^{\prime }}_{v0}$ for: (**a**) sandy silts (1.2 < $I_{D}$ < 1.8, 2.05 < $I_{c}$ < 2.95); (**b**) silty sand (1.8 < $I_{D}$ < 3.3, $I_{c}$ < 2.05).

Figure 9 and Figure 10 show the same data divided into soil type groups for clayey silt, silt, sandy silts and silty sands soils.

**Figure 11:**
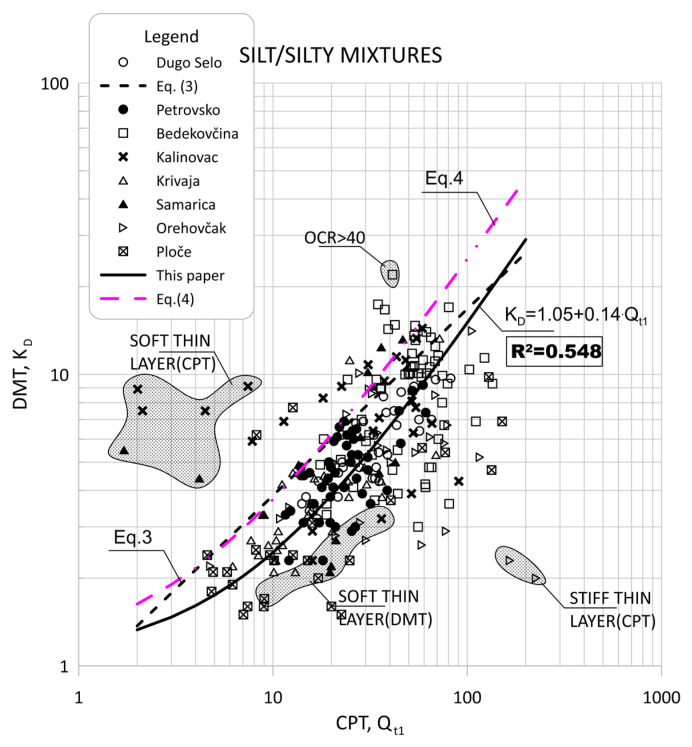
Summary of measured values from adjacent CPT and DMT profiles of $Q_{t1}$ versus $K_{D}$ for silt and silty mixtures (0.6 < $I_{D}$ < 1.8, 2.05 < $I_{c}$ < 2.95).

**Figure 12:**
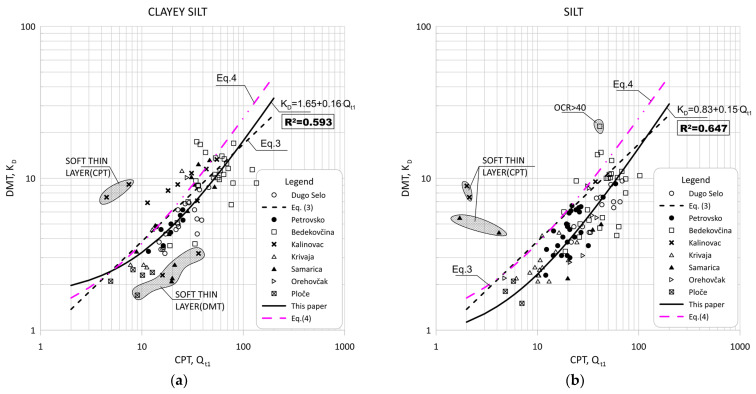
Summary of measured values from adjacent CPT and DMT profiles of $Q_{t1}$ versus $K_{D}$ for: (**a**) clayey silt (0.6 < $I_{D}$ < 0.80, 2.05 < $I_{c}$ < 2.95); (**b**) silt (0.8 < $I_{D}$ < 1.2, 2.05 < $I_{c}$ < 2.95).

**Figure 13:**
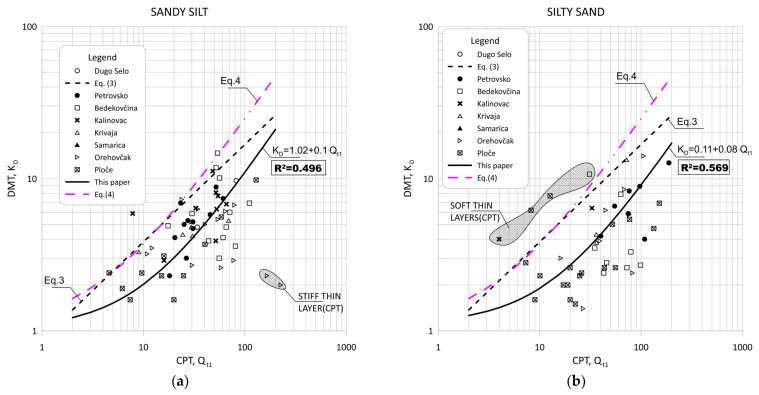
Summary of measured values from adjacent CPT and DMT profiles of $Q_{t1}$ versus $K_{D}$ for: (**a**) sandy silt (1.2 < $I_{D}$ < 1.8, 2.05 < $I_{c}$ < 2.95); (**b**) silty sand (1.8 < $I_{D}$ < 3.3, $I_{c}$ < 2.05).

Figure 11 show the data obtained for $Q_{t1}$ versus $K_{D}$ based on measurement at eight sites for silt and silty mixtures. Figure 12 and Figure 13 show the same data divided into soil type groups for clayey silt, silt, sandy silts and silty sands soils.

In general, the scatter of two different data ranges can be seen in Figure 8 through Figure 13. Both areas represent thin layers within a larger soil deposit of a different soil type. For example, the thin, soft layer area was characterized by a low resistance of the CPT probe ($q_{c}$ = 0.1–0.3 MPa), which was not confirmed by the dilatometer modulus $E_{D}$, which shows higher values ($E_{D}$ = 10–12 MPa).

The area of thin hard layers ($q_{c}$ = 15–20 MPa) was within a larger sandy soil layer, and this variation can be attributed to significant changes in the consistency of the profile (e.g., relative density and grain characteristics).

Figure 8, Figure 9, Figure 11 and Figure 12 clearly show the different behaviors of sandy soil deposits (sandy silts and silty sands). The scatter of the measured data was larger due to the different soil stratigraphy and consistency, as many sites are not uniform. All of these data were excluded from the statistical analysis.

## 6. Soil Type

**Figure 14:**
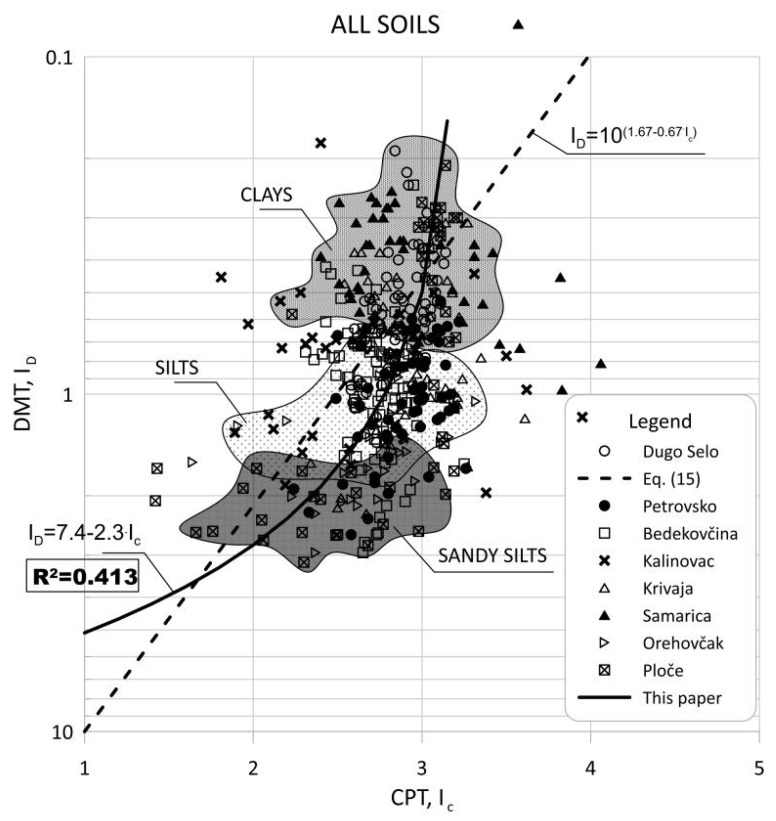
Summary of measured values from adjacent CPT and DMT profiles of $I_{D}$ versus $I_{C}$.

The DMT *I_D_* and CPT $I_{c}$ were used to identify the soil type. Figure 14 shows a summary of the measured datasets in terms of log $I_{D}$ versus $I_{c}$ without excluding any data from the statistical analysis.

Robertson [8] presented CPT and DMT data from published records and showed a trend between $I_{D}$ and $I_{c}$_,_ so it can be defined using the following relationship:

(15) $$I_{D}=10^{\left(1.67-0.67\cdot I_{c}\right)}$$

A comparison of readings from nearby in-situ test profiles at the same depth is shown in Figure 14 and reveals considerable scatter due to differences in soil stratigraphy and consistency, as many sites are not uniform. Therefore, adjacent in situ test data from the same depth did not always represent the same soil. Sandy deposits varied greatly, and individual data points from nearby in situ tests showed greater scatter.

## 7. Discussion

With the above observations as a framework, obtained correlations are given for the principal soil groups: clay-like and silty-like soils. In contrast to Robertson [8], the main soil groups were more finely subdivided in this paper, as shown in Table 4. The dilatometric modulus ($E_{D}$) and horizontal stress index ($K_{D}$) in clay soils gave correlations very similar to the published correlations (Equations (3), (4) and (6)). However, the correlations obtained in silty soils differed significantly from those published in the literature (Equations (3) and (4)). Correlations from the literature tend to underestimate *E_D_* and overestimate *K_D_* in silty soils.

### Application of Correlations for Calculating M′_DMT_

The flat dilatometer test is well known for its ability to calculate settlements of shallow foundations in soil types ranging from clay to silt and sand. Dilatometric modulus obtained with the DMT probe can be considered as a reference value for the calibration of CPTu results [14].

The proposed CPT correlations from Table 4 can be applied to obtain a constrained modulus from CPT measurements that can be used in the same procedure as the DMT. The weakest link in the process is the use of the identification parameter $I_{D}$, which corresponds poorly to the CPT identification parameter $I_{C}$. If available, much better results can be obtained by classifying the soil by a laboratory identification procedure and using the results to select the correct correlation from Table 4.

The measured dilatometer modulus ($E_{D}$) is converted to a constrained modulus (*M′_DMT_*) according to the procedure established by Marchetti [11]. As part of Marchetti’s procedure for determining the constrained modulus, the dilatometer material index ($I_{D}$) and the horizontal stress index ($K_{D}$) were also required to obtain the modulus ratio ($R_{M}$) from Table 5. The DMT constrained modulus (*M′_DMT_*) can be derived from:

(16) $${M^{\prime }}_{DMT}=R_{M}\cdot E_{D}$$

The modulus ratio $R_{M}$ is defined for different soil types, thus for the $I_{D}$ index ranges.

**Figure 15:**
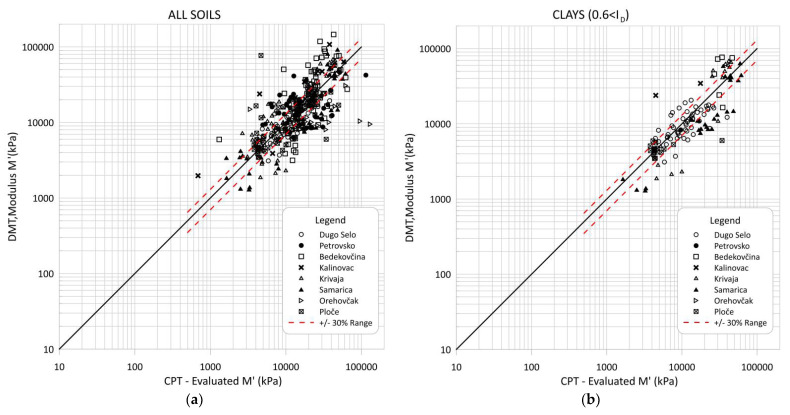
Comparison of CPT evaluated M’ through correlations and constrained modulus *M′_DMT_* from dilatometer data: (**a**) all soils; (**b**) clays.

**Figure 16:**
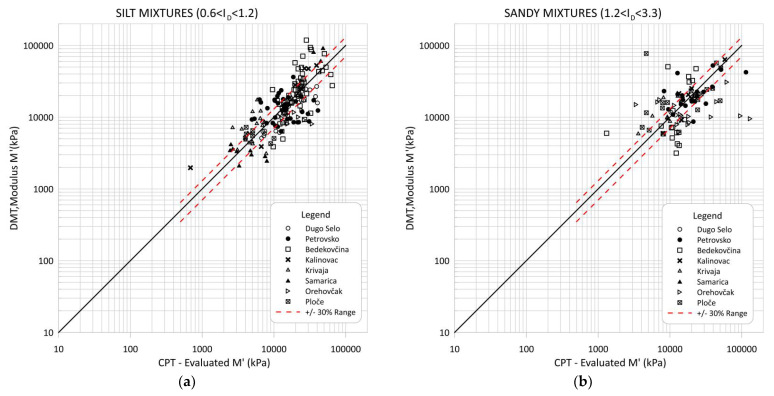
Comparison of CPT evaluated M′ through correlations and constrained modulus *M′_DMT_* from dilatometer data: (**a**) for silty mixtures; (**b**) for sandy mixtures.

Figure 15 and Figure 16 compare the modulus *M′* evaluated from CPT data based on the proposed correlations from this article with the modulus *M′_DMT_* obtained directly from the DMT test and Equation (16). Figure 15 and Figure 16 show comparisons for all soils and separately for the clay, silt and sand mixture groups. Some scattering of the obtained data can be seen, especially for sandy soils.

Most of the data were within ±30 percent error. The scattered data were the result of an error in the correlations because their inclusion did not fit well within the larger uniform soil deposits, i.e., thin intermediate soil layers were not excluded effectively from the data pool. Highly micro-structured soils could also be recognized in the interpretations and cemented aged or fissured clays. These materials can be recognized by a parameter called modified normalized small-strain rigidity index $K_{g}^{*}$ [30,31].

**Figure 17:**
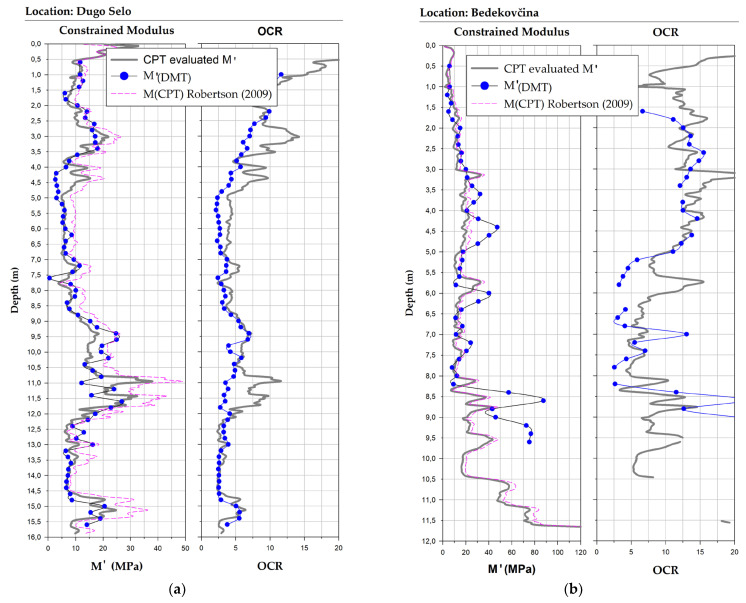
Vertical profiles of the comparison of CPT-evaluated *M′* vs. measured *M′_DMT_* and *M(CPT)* Robertson, side diagram is for *OCR_CPT_* vs. *OCR_DMT_* for the pair of in-situ probes. Continuous grey line on the chart is a result for the CPT-evaluated M’; the blue dotted line is obtained from DMT data, pink dashed line is the M(CPT) from expression by Robertson: (**a**) for the site—Dugo Selo; (**b**) for the site—Bedekovčina.

Examples of vertical profiles of the comparison between the CPT evaluated *M′* and measured *M′_DMT_* 1D constrained modulus for two of the study sites are shown in Figure 17. The constrained dilatometer modulus *M′* was calculated from the data from CPT using the correlations presented in this paper (Table 4) and applying them to the constrained dilatometer modulus Equation (16).

The additional pink line of comparison in the diagram in Figure 17 represents the constrained modulus derived from the well-established and most commonly used expression for CPT by Roberson [5]:

(17) $$M\left(CPT\right)=\alpha_{M}\left(q_{t}-\sigma_{v0}\right)$$

The constrained modulus cone factor $\alpha_{M}$ in Equation (17) was defined for different ranges of the normalized cone resistance $Q_{tn}$, also for the range of the behavior type index $I_{c}$ as shown in Table 6.

It can be seen from Figure 17 that the general overlap was better at lower OCR values and in homogeneous soil intervals. Figure 17 also shows that the local correlations are more accurate in silty soils, where the general expressions show a more significant discrepancy. An example of this is shown in the same figure for two sites: Dugo Selo in silty material and Bedekovčina in clayey material.

## 8. Conclusions

A detailed set of correlations linking the DMT parameters ($I_{D}$, $K_{D}$ and $E_{D}$) with the normalized CPT parameter ($Q_{t1}$) is proposed. Correlations were established for a refined set of mixed soils classified into narrower groups for materials classified as silty soils into four groups (silt, clayey silt, sandy silt and silty mixtures) and as clayey soils into three groups (clay, silty clay and clayey mixtures).

In each correlation plot, the corresponding published curves are plotted alongside the obtained correlation-regression equation. The obtained relationships differ greatly for different soil types, which is also evident when compared to the published correlations. The correlations published in the literature agree better for clay soils, but a significant discrepancy was observed for silty soils. From the research conducted, it is evident that transitional soils are highly influenced by a number of factors, but some of them are due to a regional soil character. It was also confirmed that the standard CPT procedure overestimates the constrained modulus in transitional soils. In general, more conservative values are recommended for use in engineering practice. Specific relationships have been revealed in silty transitional soils that serve as a guide for evaluating the behavior of mixed soils.

There is a rather large difference in the determination of the *I_D_* parameter from this paper and the Robertson expression (15). The regression curve also showed large deviations and represented the weak link of the obtained correlations. For this reason, it is recommended to use a laboratory classification procedure when choosing the appropriate correlation equation. Further research should focus on establishing a better correlation to the identification factors $I_{D}$ and $I_{C}$, which had the poorest correlation in this study. Improving these factors would increase the accuracy of the module correlations.

The comparison of the constrained modulus shows the applicability of proposed correlations and practical applicability for the settlement calculation based on CPT measurements through the DMT procedure. This is a bulky procedure at this stage, but very valuable when used as a validation procedure for the results of CPT. A continuation of this study would be to define a direct procedure that establishes the ranges of the required CPT modulus cone factors $\alpha_{M}$ for the refined soil types. The study has shown that transitional soils cannot be categorized as a broader soil group.

Although the two compared in-situ probes stress the soil at different stress levels, the intercorrelations between CPT and DMT have shown that the framework set is justified. It can also be applied in daily practice and extended to other parameters. For example, to improve the accuracy in future investigations in mixed silty to sandy soils, it is necessary to introduce new parameters related to the CPT-normalized pore pressure. The proposed equations are based on local geological formations and can be used as a valuable guide for a local site-specific investigation.

## Figures and Tables

**Table 1:** Technical specifications of the CPT Icone digital cone.

“Icone” Digital Cone	Technical Characteristics	Maximum Range
Resolution	24 bits (*Ix*/*Iy* 16 bits)	
Cone tip area	10 cm^2^	
Available parameters	$q_{c}$$,\;f_{s}$$,\;u_{2}$, *Ix*/*Iy*	
Memo function	16 Mbit (8 hrs. CPT operation)	
Real-time data processing		
Cone resistance ($q_{c}$)	75 MPa	150 MPa
Minimal accuracy for Class 2	100 kPa or 5%	
Sleeve friction ($f_{s}$)	1 MPa	1.5 MPa
Minimal accuracy for Class 2	15 kPa od 3%	
Pore water pressure (*u*)	2 MPa	3 MPa
Minimal accuracy for Class 2	25 kPa od 2%	
Inclination (*Ix*/*Iy*)	20°	25°
Minimal accuracy for Class 2	2°	

**Table 2:** List of study sites including measured DMT profiles details.

No.	Site Name	Soil Type	Reached Depth (m)	$I_{D}$ Parameter Range (DMT)		$K_{D}$ Parameter Range (DMT)		$E_{D}/{\sigma^{\prime }}_{v0}$ Range (DMT)	
1	Dugo Selo	soft clays to soft silty-clay and silts	0–15.6	0.1–1.4	(s = 0.26)	3.2–10	(s = 1.50)	6–470	(s = 76)
2	Petrovsko	silts to silty sands	0–6.8	0.6–2.6	(s = 0.48)	2.0–13	(s = 1.30)	64–990	(s = 110)
3	Bedekovčina	clayey silts to silty sands	0–9.6	0.4–3.6	(s = 0.61)	2.4–22	(s = 4.30)	84–776	(s = 110)
4	Kalinovac	clayey silts to silty sand	0–7.2	0.4–2.0	(s = 0.66)	2.9–19	(s = 4.20)	52–648	(s = 132)
5	Krivaja	silty clays to silty sands	0–9.2	0.3–2.3	(s = 0.49)	1.7–14	(s = 4.20)	26–970	(s = 142)
6	Samarica	soft clays to silts	0–10	0.25–1.0	(s = 0.20)	1.5–14	(s = 3.40)	5–316	(s = 74)
7	Orehovčak	silts to silty sands	0–6.6	0.75–3.0	(s = 1.22)	1.4–14	(s = 2.80)	78–1441	(s = 322)
8	Ploče	soft marine clays to silty sands	5.6–16	0.2–3.2	(s = 0.90)	1.5–10	(s = 1.60)	21–565	(s = 149)

**Table 3:** List of study sites including measured CPT profiles details.

No.	Site Name	Soil Type	Reached Depth (m)	$Q_{t1}$ Parameter Range (CPT)		$I_{c}$ Parameter Range (CPT)	
1	Dugo Selo	soft clays to soft silty-clay and silts	0–15.6	9–82	(s = 15)	2.6–3.1	(s = 0.17)
2	Petrovsko	silts to silty sands	0–6.8	12–187	(s = 20)	2.1–3.2	(s = 0.21)
3	Bedekovčina	clayey silts to silty sands	0–9.6	18–134	(s = 20)	2.3–3.2	(s = 0.14)
4	Kalinovac	clayey silts to silty sand	0–7.2	2–67	(s = 23)	1.8–3.9	(s = 0.52)
5	Krivaja	silty clays to silty sands	0–9.2	6–72	(s = 14)	2.5–3.6	(s = 0.27)
6	Samarica	soft clays to silts	0–10	2–52	(s = 13)	2.4–4.0	(s = 0.38)
7	Orehovčak	silts to silty sands	0–6.6	5–226	(s = 47)	1.6–3.3	(s = 0.35)
8	Ploče	soft marine clays to silty sands	5.6–16	4–151	(s = 33)	1.4–3.2	(s = 0.49)

**Table 4:** Proposed correlations for clay-like and silty-like soils.

Soil Type	Parameter	Regression
Clay and clays mixtures	$E_{D}/{\sigma^{\prime }}_{v0}=7.25\cdot {\left(Q_{t1}\right)}^{0.84}$	$R^{2}=0.713$
	$K_{D}=2.06+0.17\cdot Q_{t1}$	$R^{2}=0.731$
Clay	$E_{D}/{\sigma^{\prime }}_{v0}=11.62\cdot {\left(Q_{t1}\right)}^{0.55}$	$R^{2}=0.851$
	$K_{D}=1.85+0.2\cdot Q_{t1}$	$R^{2}=0.853$
Silty clay	$E_{D}/{\sigma^{\prime }}_{v0}=7.17\cdot {\left(Q_{t1}\right)}^{0.87}$	$R^{2}=0.662$
	$K_{D}=2.04+0.17\cdot Q_{t1}$	$R^{2}=0.693$
Silt and silty mixtures	$E_{D}/{\sigma^{\prime }}_{v0}=22.31\cdot {\left(Q_{t1}\right)}^{0.64}$	$R^{2}=0.653$
	$K_{D}=1.05+0.14\cdot Q_{t1}$	$R^{2}=0.548$
Clayey silt	$E_{D}/{\sigma^{\prime }}_{v0}=12.85\cdot {\left(Q_{t1}\right)}^{0.73}$	$R^{2}=0.612$
	$K_{D}=1.65+0.16\cdot Q_{t1}$	$R^{2}=0.593$
Silt	$E_{D}/{\sigma^{\prime }}_{v0}=22.31\cdot {\left(Q_{t1}\right)}^{0.64}$	$R^{2}=0.653$
	$K_{D}=0.83+0.15\cdot Q_{t1}$	$R^{2}=0.647$
Sandy silt	$E_{D}/{\sigma^{\prime }}_{v0}=47.52\cdot {\left(Q_{t1}\right)}^{0.47}$	$R^{2}=0.467$
	$K_{D}=1.02+0.1\cdot Q_{t1}$	$R^{2}=0.496$
Silty sand	$E_{D}/{\sigma^{\prime }}_{v0}=49\cdot {\left(Q_{t1}\right)}^{0.49}$	$R^{2}=0.291$
	$K_{D}=0.11+0.08\cdot Q_{t1}$	$R^{2}=0.569$
All soils	$I_{D}=7.4-2.3\cdot I_{c}$	$R^{2}=0.413$

**Table 5:** Constrained modulus parameter ($R_{M}$) for settlement calculations.

Conditions	Relationship for $R_{M}$	Notes
$I_{D}<0.6$	$R_{M}=0.14+2.36\cdot logK_{D}$	clay soils
$I_{D}>3$	$R_{M}=0.50+2.0\cdot logK_{D}$	clean Sands
$0.6<I_{D}<3$	$R_{M}=R_{Mo}\left(2.5-R_{Mo}\right)\cdot logK_{D}$	silts to silty sands
	$R_{Mo}=0.14+0.15\cdot \left(I_{D}-0.6\right)$	
$if\;K_{D}>10$	$R_{M}=0.32+2.18\cdot logK_{D}$	
$if\;R_{M}<0.85$	$set\;R_{M}=0.85$	

**Table 6:** Constrained modulus factor $\alpha_{M}$, as defined by Robertson.

Conditions		Relationship for $\alpha_{M}$	Notes
$I_{c}>2.2\;$	$Q_{tn}<14$	$\alpha_{M}=Q_{tn}$	fine-grained soil (soft clay)
$I_{c}>2.2\;$	$Q_{tn}>14$	$Q_{tn}=14$	stiff fine-grained soil
$I_{c}<2.2\;$		$\alpha_{M}=0.03\cdot 10^{\left(0.55I_{c}+1.68\right)}$	coarse-grained soils

## Data Availability

Data sharing not applicable.
